# Supplementary material for: Multi‐dataset identification of innovative feature genes and molecular mechanisms in keratoconus
Source: J Cell Mol Med. 2024 Sep 19;28(18):e70079. doi: 10.1111/jcmm.70079 (PMC11412914; doi:10.1111/jcmm.70079)
Supplement: Supplementary file 2 — Material S2. [file JCMM-28-e70079-s002.zip › 7.GSEA analysis/5.RNF217-AS1/KEGG_EPITHELIAL_CELL_SIGNALING_IN_HELICOBACTER_PYLORI_INFECTION.html]

Details for gene set KEGG\_EPITHELIAL\_CELL\_SIGNALING\_IN\_HELICOBACTER\_PYLORI\_INFECTION[GSEA]

|  || Dataset | RNF217-AS1.group.cls#h\_versus\_l |
| Phenotype | group.cls#h\_versus\_l |
| Upregulated in class | l |
| GeneSet | KEGG\_EPITHELIAL\_CELL\_SIGNALING\_IN\_HELICOBACTER\_PYLORI\_INFECTION |
| Enrichment Score (ES) | -0.3860566 |
| Normalized Enrichment Score (NES) | -1.3421715 |
| Nominal p-value | 0.16829745 |
| FDR q-value | 0.36303186 |
| FWER p-Value | 0.924 |
Table: GSEA Results Summary

  

Fig 1: Enrichment plot: KEGG\_EPITHELIAL\_CELL\_SIGNALING\_IN\_HELICOBACTER\_PYLORI\_INFECTION      
 Profile of the Running ES Score & Positions of GeneSet Members on the Rank Ordered List

  

| SYMBOL | TITLE | RANK IN GENE LIST | RANK METRIC SCORE | RUNNING ES | CORE ENRICHMENT || 1 | ATP6V0D2 | na | 2490 | 0.317 | -0.0262 | No |
| 2 | IKBKB | na | 4368 | 0.238 | -0.0459 | No |
| 3 | CXCR2 | na | 5883 | 0.187 | -0.0627 | No |
| 4 | HBEGF | na | 8507 | 0.116 | -0.1217 | No |
| 5 | MAPK12 | na | 9095 | 0.103 | -0.1237 | No |
| 6 | ATP6V0D1 | na | 9204 | 0.101 | -0.1122 | No |
| 7 | CASP3 | na | 9912 | 0.088 | -0.1200 | No |
| 8 | GIT1 | na | 10004 | 0.086 | -0.1101 | No |
| 9 | CXCL8 | na | 10297 | 0.081 | -0.1069 | No |
| 10 | MAPK13 | na | 10953 | 0.071 | -0.1156 | No |
| 11 | TJP1 | na | 12076 | 0.054 | -0.1401 | No |
| 12 | MAPK11 | na | 12121 | 0.054 | -0.1336 | No |
| 13 | ADAM17 | na | 12204 | 0.053 | -0.1284 | No |
| 14 | ADAM10 | na | 12664 | 0.047 | -0.1349 | No |
| 15 | PTPN11 | na | 12805 | 0.045 | -0.1325 | No |
| 16 | ATP6V1G3 | na | 12812 | 0.045 | -0.1262 | No |
| 17 | MAPK9 | na | 12895 | 0.044 | -0.1223 | No |
| 18 | F11R | na | 13197 | 0.040 | -0.1253 | No |
| 19 | SRC | na | 13502 | 0.036 | -0.1289 | No |
| 20 | PLCG1 | na | 13642 | 0.035 | -0.1279 | No |
| 21 | MAPK14 | na | 14311 | 0.027 | -0.1433 | No |
| 22 | ATP6V0B | na | 15585 | 0.013 | -0.1781 | No |
| 23 | MAPK10 | na | 15620 | 0.013 | -0.1773 | No |
| 24 | ATP6V1D | na | 15821 | 0.011 | -0.1815 | No |
| 25 | MAP2K4 | na | 16303 | 0.006 | -0.1946 | No |
| 26 | ATP6V0E1 | na | 16390 | 0.005 | -0.1964 | No |
| 27 | CHUK | na | 16613 | 0.003 | -0.2024 | No |
| 28 | MAPK8 | na | 16681 | 0.002 | -0.2040 | No |
| 29 | CDC42 | na | 18054 | -0.011 | -0.2420 | No |
| 30 | CSK | na | 18243 | -0.013 | -0.2455 | No |
| 31 | ATP6V1F | na | 18311 | -0.014 | -0.2455 | No |
| 32 | RAC1 | na | 18357 | -0.014 | -0.2448 | No |
| 33 | ATP6V0C | na | 18707 | -0.017 | -0.2523 | No |
| 34 | ATP6V1G1 | na | 19140 | -0.021 | -0.2617 | No |
| 35 | ATP6V1C1 | na | 19502 | -0.025 | -0.2685 | No |
| 36 | ATP6V1B2 | na | 19557 | -0.026 | -0.2663 | No |
| 37 | ATP6V1E1 | na | 20165 | -0.032 | -0.2793 | No |
| 38 | ATP6V1A | na | 20315 | -0.033 | -0.2788 | No |
| 39 | ATP6AP1 | na | 20488 | -0.035 | -0.2788 | No |
| 40 | NFKB1 | na | 20531 | -0.035 | -0.2749 | No |
| 41 | IKBKG | na | 20686 | -0.037 | -0.2741 | No |
| 42 | ATP6V1C2 | na | 21292 | -0.043 | -0.2853 | No |
| 43 | RELA | na | 21550 | -0.046 | -0.2861 | No |
| 44 | ATP6V1H | na | 21597 | -0.046 | -0.2808 | No |
| 45 | ATP6V0A1 | na | 21733 | -0.048 | -0.2778 | No |
| 46 | MET | na | 22571 | -0.057 | -0.2938 | No |
| 47 | PAK1 | na | 23429 | -0.067 | -0.3088 | No |
| 48 | NOD1 | na | 23431 | -0.067 | -0.2991 | No |
| 49 | EGFR | na | 24191 | -0.078 | -0.3098 | No |
| 50 | NFKBIA | na | 24662 | -0.085 | -0.3111 | No |
| 51 | PLCG2 | na | 24806 | -0.087 | -0.3026 | No |
| 52 | LYN | na | 24892 | -0.089 | -0.2923 | No |
| 53 | JUN | na | 27259 | -0.131 | -0.3416 | No |
| 54 | IGSF5 | na | 27351 | -0.133 | -0.3251 | No |
| 55 | MAP3K14 | na | 28008 | -0.149 | -0.3226 | No |
| 56 | ATP6V1E2 | na | 30210 | -0.213 | -0.3553 | Yes |
| 57 | ATP6V0A4 | na | 30469 | -0.223 | -0.3307 | Yes |
| 58 | JAM2 | na | 30879 | -0.237 | -0.3083 | Yes |
| 59 | CXCR1 | na | 31763 | -0.277 | -0.2939 | Yes |
| 60 | PTPRZ1 | na | 32817 | -0.346 | -0.2743 | Yes |
| 61 | CCL5 | na | 32851 | -0.349 | -0.2250 | Yes |
| 62 | ATP6V1G2 | na | 32923 | -0.355 | -0.1759 | Yes |
| 63 | ATP6V1B1 | na | 32937 | -0.356 | -0.1250 | Yes |
| 64 | ATP6V0E2 | na | 33133 | -0.370 | -0.0773 | Yes |
| 65 | CXCL1 | na | 33320 | -0.387 | -0.0268 | Yes |
| 66 | JAM3 | na | 33927 | -0.468 | 0.0231 | Yes |
Table: GSEA details [plain text format]

  

Fig 2: KEGG\_EPITHELIAL\_CELL\_SIGNALING\_IN\_HELICOBACTER\_PYLORI\_INFECTION      
 Blue-Pink O' Gram in the Space of the Analyzed GeneSet

  

Fig 3: KEGG\_EPITHELIAL\_CELL\_SIGNALING\_IN\_HELICOBACTER\_PYLORI\_INFECTION: Random ES distribution      
 Gene set null distribution of ES for **KEGG\_EPITHELIAL\_CELL\_SIGNALING\_IN\_HELICOBACTER\_PYLORI\_INFECTION**
